# Mesoscale Process Modeling of a Thick Pultruded Composite with Variability in Fiber Volume Fraction

**DOI:** 10.3390/ma14133763

**Published:** 2021-07-05

**Authors:** Onur Yuksel, Michael Sandberg, Jesper H. Hattel, Remko Akkerman, Ismet Baran

**Affiliations:** 1 Faculty of Engineering Technology, University of Twente, NL-7500 AE Enschede, The Netherlands; o.yuksel@utwente.nl (O.Y.); r.akkerman@utwente.nl (R.A.); 2 Department of Mechanical Engineering, Technical University of Denmark, DK-2800 Kgs. Lyngby, Denmark; ms@ece.au.dk (M.S.); jhat@mek.dtu.dk (J.H.H.); 3 Department of Electrical and Computer Engineering, Aarhus University, DK-8200 Aarhus N, Denmark

**Keywords:** pultrusion, fiber volume fraction, nonuniformity, mesoscale, process modeling, residual stress

## Abstract

Pultruded fiber-reinforced polymer composites are susceptible to microstructural nonuniformity such as variability in fiber volume fraction ($V_{f}$), which can have a profound effect on process-induced residual stress. Until now, this effect of non-uniform $V_{f}$ distribution has been hardly addressed in the process models. In the present study, we characterized the $V_{f}$ distribution and accompanying nonuniformity in a unidirectional fiber-reinforced pultruded profile using optical light microscopy. The identified nonuniformity in $V_{f}$ was subsequently implemented in a mesoscale thermal–chemical–mechanical process model, developed explicitly for the pultrusion process. In our process model, the constitutive material behavior was defined locally with respect to the corresponding fiber volume fraction value in different-sized representative volume elements. The effect of nonuniformity on the temperature and cure degree evolution, and residual stress was analyzed in depth. The results show that the nonuniformity in fiber volume fraction across the cross-section increased the absolute magnitude of the predicted residual stress, leading to a more scattered residual stress distribution. The observed $V_{f}$ gradient promotes tensile residual stress at the core and compressive residual stress at the outer regions. Consequently, it is concluded that it is essential to take the effects of nonuniformity in fiber distribution into account for residual stress estimations, and the proposed numerical framework was found to be an efficient tool to study this aspect.

## 1. Introduction

Composite materials’ structure governs their effective properties, and the structure is governed by the processing steps during manufacture [1]. Here, the fiber arrangement impacts several properties of the composite part (e.g., electrical, thermal, and mechanical) as well as process-induced effects. The mechanical performance, which is considered a mechanical property, is directly influenced by the process-induced residual stress [2,3]. Therefore, the coupled nature of processing, structure, and residual stress gives critical information about composite materials’ performance. In this paper, the relationship between the meso-structure (associated with the fiber arrangement) and residual stress in a pultruded profile is the main focus.

Pultrusion is an increasingly popular production technique to manufacture fiber-reinforced composite profiles [4]. The process is a highly efficient way to produce composite profiles due to its continuous and automatized nature. In a pultrusion process, the resin impregnated fiber reinforcement is pulled through a heated die to produce constant cross-sectional composite profiles [5].

Pultruded profiles are subject to residual stresses due to non-uniform heating/cooling, non-uniform fiber distribution, cure shrinkage, coefficient of thermal expansion mismatch, elastic moduli evolution gradient through the cross-section, resulting in geometrical distortions [6,7,8], and pre-mature cracks [9]. The locked in residual stresses should be taken into account to prevent unprecedented failure or undesirable product rejection due to geometrical distortions [2]. Since the cure reaction takes place while the profile is moving during the pultrusion process, a specialized numerical framework is needed to analyze pultrusion. This is a subject that has been studied extensively in the literature.

In one of the earliest works, a thermo–chemical model of pultrusion was carried out with a 1D Lagrangian model that followed the profile throughout the process [10]. Later, full 2D and 3D methods were developed for thermo–chemical modeling [11,12,13]. The impregnation flow of the resin system has also been coupled with thermo–chemical models [14]. This analysis is essential for resin injection pultrusion. In [15,16], 2D and 3D mechanical models in the Lagrangian frame were coupled with a 3D thermo–chemical model in the Eulerian frame to predict the residual stress formation in pultrusion. Accordingly, in a recent paper, a fully coupled 3D Eulerian model was developed [17]. Using these approaches, numerous studies have been carried out in the literature to predict the residual stress in pultruded components with different cross-sectional shapes, reinforcement configurations, and for different process parameters [6,7,9,15,18,19]. These numerical models showed that the residual stress locked in a pultruded profile could reach critical levels for the failure initiation and the profile’s ultimate strength.

The process models of process-induced stress in pultrusion have been validated experimentally. For example, the spring-in of an L-shaped pultruded profile and warpage in a hollow pultruded profile were analyzed numerically and experimentally in [6,7], respectively. In later research, Ref. [20] reported the spring-in values of L-shaped profiles produced with different pulling speeds. In addition to shape distortions, experimental validation of process-induced stresses has been conducted as well. The tensile residual stress at the core of a thick square profile was validated via hole drilling experiments in [21,22]. Here it was found that the measured and the predicted residual stress values can reach up to 10–15% of the estimated transverse tensile strength. Therefore, it was argued that it is essential to consider the residual stress in the initial design steps.

**Figure 1:**
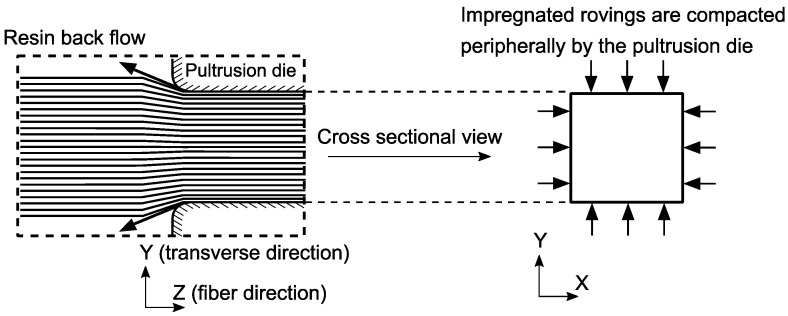
Schematic representation of the peripheral compaction.

The structural variability should also be considered in the design steps as $V_{f}$ is one of the most prominent parameters defining the performance of a fiber-reinforced polymer composite (FRPC). The structural variability in FRPC can be found in the form of fiber waviness, misalignment, wrinkling, intra and inter-tow resin-rich regions, etc. [23,24,25,26]. Especially in thick composites, consolidation results in ply thickness variability, which causes fiber volume fraction ($V_{f}$) variability in the through thickness direction [27]. The internal variability in fiber distribution has also consequences on residual stress and process-induced deformations. For example, in [28], a $V_{f}$ gradient was introduced by a resin bleeder, which resulted in spring-in of a composite laminate. The effect of fiber wrinkling on the spring-in value was shown in [29]. Pultruded composites have structural variability as well. The $V_{f}$ values from different locations within I-shaped pultruded profiles were observed via optical microscopy and image processing in [30], and the fiber distribution was reported as neither uniform nor totally random. Local and global variability of fiber volume fraction in pultruded profiles differs for the reinforcement type or geometry of the profile [31,32]. In the resin bath pultrusion, the resin impregnated rovings are consolidated by a solid die and the excess resin is bleed out via squeeze flow [33]. This peripheral compaction, as schematically shown in Figure 1, can be hypothesized as one of the sources of $V_{f}$ gradient through the cross-section. In addition to this global $V_{f}$ variability, a micro/meso-scale variability in $V_{f}$ is inevitable due to intra-roving resin-rich layers and randomly distributed filament within the inter-roving regions.

Detailed analyses of fiber misalignment, fiber mat misalignment, resin-rich areas, and void topology in pultrusion products have been performed via optical micrography, Fourier transformation, and computational tomography [34,35,36]. In addition to the characterization of the variability in fiber distribution, its effects on a pultruded bridge deck profile’s ultimate properties/performance were investigated in [37]. The effect of non-uniform fiber distribution on the cure degree and temperature evolution through the process was studied numerically in [38]. Local $V_{f}$ variability has been implemented in using a pixel-based finite element method [39]. Here, the elastic material properties within a selected area were defined with respect to each finite element’s corresponding $V_{f}$. In our previous work [40], we have developed a simplistic model and investigated the effect of $V_{f}$ nonuniformity through the cross-section of a pultruded profile. There has been limited studies to unravel the influence of local $V_{f}$ distribution on the residual stresses for pultruded thick composites. It is a scientific challenge that requires the actual $V_{f}$ distribution of the whole cross-section of a thick pultruded composite and implementation of location dependent material properties with a proper RVE selection. Indeed, the microstructural variability through the whole structure is mostly overlooked in the literature, which leads to ruling out the effects caused by the processing-structure relationship. Moreover, to the best of the author’s knowledge, the correlation of $V_{f}$ distribution with the local residual stress evolution has not been studied yet.

In the present work, the $V_{f}$ nonuniformity in a unidirectional (UD) fiber-reinforced pultruded profile, which is a consequence of compaction at the entrance of the die as hypothesized, is analyzed in depth, and a mesoscale process modeling framework, which takes the nonuniformity into account, is demonstrated. we build upon our previous outcomes and improve our model by introducing; (i) the effect of patch size on the temperature, cure degree, and local variations of residual stress and its magnitude, (ii) location dependent residual stress concerning local $V_{f}$. We address these points by doing the following. First, the fiber distribution across the whole cross-section of a relatively thick pultruded profile is presented. The $V_{f}$ dependent thermal, chemical, and mechanical material property evolutions are calculated and implemented into a 2D Lagrangian thermo–chemical–mechanical process model. The difference in predicted temperature and cure degree fields at the exit of the die for different RVE sizes representing the fiber arrangement is presented. The evolution of residual stresses on various locations through the cross-section is inspected to reveal the influence of $V_{f}$ on the predicted residual stress. Location dependent residual stress distribution for the corresponding $V_{f}$ values is analyzed in depth. The critical regions under different loading conditions are discussed concerning processing–structure–property relationships.

## 2. Materials and Methods

**Figure 2:**
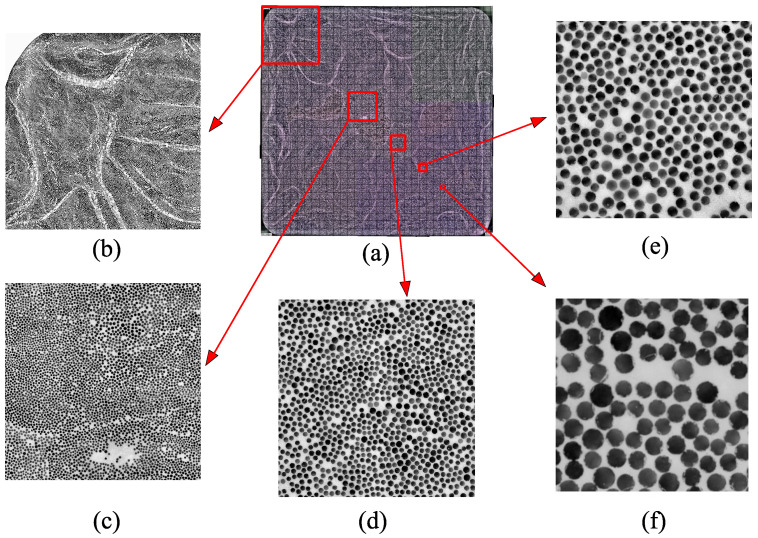
One of the analyzed cross sections (**a**) with examples of different RVE sizes for 4 × 4 patches (Case A), (**b**) 8 × 8 patches (Case B), (**c**) 16 × 16 patches (Case C), (**d**) 32 × 32 patches (Case D) (**e**) and 64 × 64 patches (Case E) (**f**). The grid used in (**a**) reflects 32 × 32 patches.

A unidirectional glass fiber-reinforced polyester-based pultruded profile (19.5 × 19.5 mm${}^{2}$) was investigated in this study. Samples were cut using a diamond saw with water cooling. Before embedding the sample into cold mounting epoxy, they were kept in a vacuum oven overnight at 45 °C. The samples were ground and polished using 500,1000,2000,4000 grit size grinding papers and 9, 3, 1 μm solutions in an automated polishing machine ‘Struers Tegramin-30’ (Struers, Cleveland, OH, USA). An optical microscope, ‘Keyence VHX 1000’ (Keyence, Osaka, Japan), was used to capture the micrographs. Ring illumination was used during micrography as it results in higher contrast due to the opaque resin system and transparent glass fibers. Images were taken with 400× magnification with automatic stitching. The stitched images taken from the microscope were stitched again manually to yield a single image of the whole cross-sectional area of 19.5 × 19.5 mm${}^{2}$. The investigated cross-sectional area can be seen in Figure 2a.

The $V_{f}$ distribution through the cross-section was calculated using an image processing tool in Matlab (R2019b). First, individual fibers were detected via the circle finding function in Matlab (R2019b), ‘imfindcircles’. Subsequently, overlapped circles were identified and eliminated using an in-house code. The $V_{f}$ was calculated from the area fraction of fibers to the total area. The average fiber volume fraction values were calculated for different-sized RVEs throughout the cross-section. A complete cross-sectional view of the sample together with RVEs of the selected dimensions can be seen in Figure 2. The labels used to represent each RVE size case and the corresponding number of patches through the cross-section together with the RVE edge lengths can be seen in Table 1.

## 3. Numerical Modeling Framework

**Figure 3:**
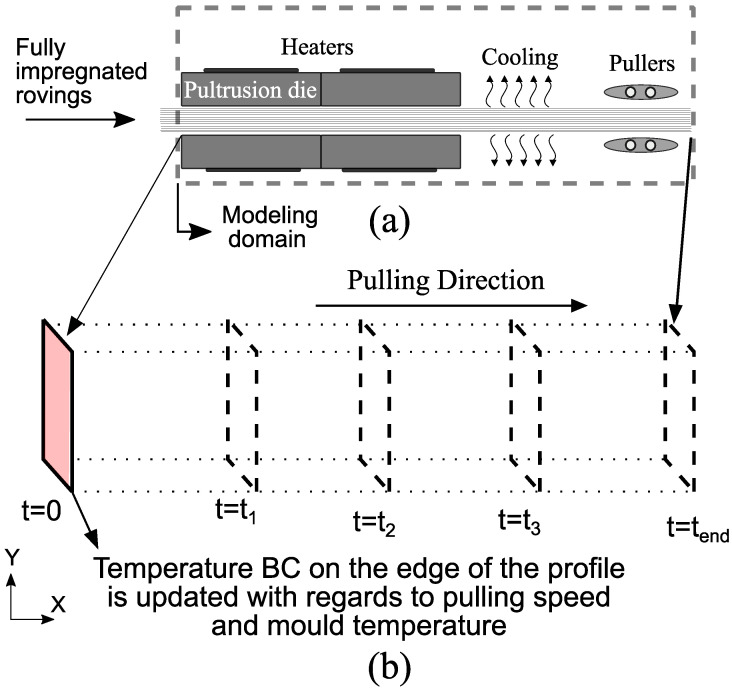
(**a**) Schematic representation of a pultrusion line and the modeling domain. (**b**) Schematic representation of the modeling framework.

A 2D Lagrangian modeling framework was used to solve the coupled thermo– chemical– mechanical problem in the present study. In this modeling approach, a 2D material frame, representing a cross-section of the profile, moves along the pulling direction starting from the die entrance until the profile is cooled down. Similar to [10], the heat transfer in the fiber direction was neglected. This simplified 2D model allows using a fine mesh discretization, as it yields a fast computation time. This is essential to implement the $V_{f}$ variability with a high resolution throughout the cross-section, which is why this approach was chosen. A schematic representation of the modeling framework can be seen in Figure 3.

As seen in Figure 3, a temperature boundary condition was implemented on the outer surfaces of the profile. In other words, the equations were only solved for the composite material. The 2D transient heat conduction equation is given in Equation (1).

(1) $$\begin{array}{c} \rho_{c}Cp_{c}\frac{\partial T}{\partial t}=k_{X}\frac{\partial^{2}T}{\partial X^{2}}+k_{Y}\frac{\partial^{2}T}{\partial Y^{2}}+q \end{array}$$

where $\rho_{c}$ is the density, $Cp_{c}$ is the specific heat capacity of the composite, *T* is the temperature, *t* is time, *X* and *Y* are the in-plane directions, and $k_{X}=k_{Y}$ are the thermal conductivities. Finally, *q* is the heat source term coming from the exothermic heat reaction of thermoset resin.

A 2D generalized plane strain formulation was used to estimate the displacement field of the cross-section. The incremental mechanical strain and stress components followed Equations (2) and (3).

(2) $$\begin{array}{c} \Delta \epsilon_{ij}^{mech}=\Delta \epsilon_{ij}^{tot}-\Delta \epsilon_{ij}^{therm}-\Delta \epsilon_{ij}^{chem} \end{array}$$

(3) $$\begin{array}{c} \Delta \sigma_{ij}=J\Delta \epsilon_{ij}^{mech} \end{array}$$

where $\Delta \epsilon_{ij}^{mech}$, $\Delta \epsilon_{ij}^{tot}$, $\Delta \epsilon_{ij}^{therm}$ and $\Delta \epsilon_{ij}^{chem}$ are the mechanical, total, thermal and chemical strain increments, respectively. $\Delta \sigma_{ij}$ is the incremental stress component and *J* is the Jacobian matrix. The entries in *J* can be seen in Equation (23) in [15].

### 3.1. Chemical and Thermo-Mechanical Material Models of Polyester Resin

The resin’s chemical structure evolves via curing, which results in an exothermic reaction, where the physical state of resin changes from a liquid to a rubbery and then a solid state. Therefore, the temperature and cure degree dependent constitutive behavior of resin is essential in process modeling. The resin system used in this profile was characterized in [41]. The corresponding material models, namely the cure kinetics and CHILE (cure hardening instantaneous linear elastic) models, are briefly presented in this study. Readers are referred to [41,42] for detailed information about the characterization of the material systems used in pultrusion.

The cure kinetics was modeled using an autocatalytic model with an Arrhenius type temperature dependency. Therefore, the cure degree (α) evolution and heat generation terms were captured by Equation (4).

(4) $$\begin{array}{c} \frac{d\alpha }{dt}=A_{0}\exp\left(\frac{-E_{a}}{RT}\right)\alpha^{m}{\left(1-\alpha \right)}^{n} \end{array}$$

where $A_{0}$ is the pre-exponential constant, $E_{a}$ is the activation energy, *m* and *n* are the reaction orders. α is the instantaneous cure degree, *R* is the gas constant, and *T* is the absolute temperature. The total exothermic heat reaction was measured as 175±15 kJ/kg. The cure kinetics model parameters can be seen in Table 2.

A CHILE model was used to implement the cure degree and temperature dependent elastic modulus evolution in the process model. The model used in the present study is shown in Equation (5) as:

(5) $$\begin{array}{c} \;E_{r}=\left\{\begin{array}{cc} E_{r}^{0}, & T^{*}\leq T_{C1}\\ A_{e}\exp\left(K_{e}T^{*}\right), & T_{C1}<T^{*}\leq T_{C2}\\ E_{r}^{1}+\frac{T^{*}-T_{C2}}{T_{C3}-T_{C2}}\left(E_{r}^{\infty }-E_{r}^{1}\right), & T_{C2}<T^{*}\leq T_{C3}\\ E_{r}^{\infty }, & T_{C3}<T^{*} \end{array}\right.\; \end{array}$$

where $T_{C1},T_{C2},T_{C3}$ and $E_{r}^{0},E_{r}^{1},E_{r}^{\infty }$ are the critical temperatures and the associated elastic moduli, respectively. $A_{e}$ and $K_{e}$ are the constants describing an exponential rise in stiffness during cure. $T^{*}$ is equal to the difference between the instantaneous temperature and the glass transition temperature of the resin ($T^{*}=T_{g}-T$). The instantaneous glass transition temperature is modeled with a linear relationship to the degree of cure from 0 to 135 °C ($T_{g}=135\times \alpha$). The constants used in the elastic modulus model can be found in Table 3.

Other than cure kinetics and the elastic modulus evolution of resin through the process, the thermal expansion and cure shrinkage behaviors of the resin system are also essential to predicting process-induced stresses. As shown in [43], the coefficient of thermal expansion (CTE) value of a thermoset resin is generally higher above $T_{g}$ than below $T_{g}$. In this study, the CTE of polyester resin was set to 72 ppm/°C below $T_{g}$. Accordingly, the CTE in the rubbery state (above $T_{g}$) was set to 180 ppm/°C, which is 1.5 times higher than the CTE in its glassy state. In [7], experimental and numerical analysis of an L-shaped pultruded profile produced with a similar resin system showed a good agreement with the experimentally measured spring-in value when a 6% of volumetric shrinkage was chosen. Therefore, the total volumetric shrinkage value was defined as 6% in the present study.

### 3.2. Thermal and Thermo-Mechanical Constitutive Material Properties

The thermal and mechanical constitutive material properties of the composite material were calculated based on the corresponding fiber volume fraction and constituents’ properties (the glass fiber and polyester resin). The thermal and mechanical properties of the glass fiber and polyester resin used in this study are presented in Table 4.

Density, specific heat capacity, and thermal conductivity are the required properties to solve the thermal equations. The density, the specific heat capacity, and the conductivity of representative volume elements were calculated via rule of mixture as a commonly accepted and a simplistic approach for process modeling of composites. The thermal conductivity transverse to the fiber direction was calculated using the relationship in Equation (6) [44].

(6) $$k_{X}=k_{Y}=\frac{k_{r}\cdot k_{f}}{\omega_{r}\cdot k_{f}+\omega_{f}\cdot k_{r}}$$

where $k_{f}$ and $k_{r}$ are the thermal conductivity of the fibers and resin, respectively. $\omega_{f}$ and $\omega_{r}$ are the weight fractions of the fiber and resin.

The mechanical constitutive behavior, the equivalent thermal expansion/shrinkage, and the chemical shrinkage are required to estimate the evolution of process-induced stress. Here, the $V_{f}$ dependent elastic behavior of the composite as well as the thermal expansion and cure shrinkage coefficients were captured via the self-consistent field micromechanics (SCFM) by Bogetti and Gillespie [45] which is a commonly used micromechanics approach for continuous UD fiber-reinforced composites.

### 3.3. Coupled Temperature Displacement Model

**Figure 4:**
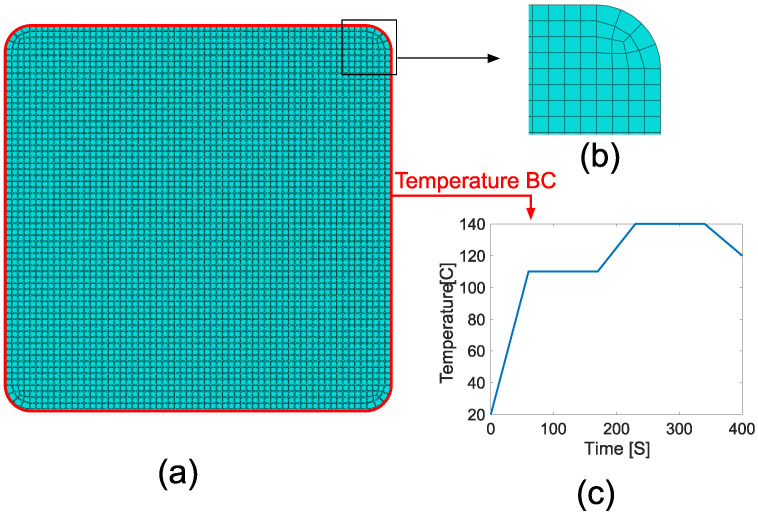
(**a**) 2D modeling domain, (**b**) a closer look at the mesh structure, (**c**) temperature profile defined on the boundary (the outer surfaces of the profile).

The coupled thermo–chemical–mechanical model was developed in Abaqus. As shown in Figure 4, a temperature boundary condition was applied to the outer nodes of the 2D Lagrangian frame that was moved along the pulling direction. The die was considered to be rigid. The interaction between the die and the composite was defined as frictionless in the tangential direction. It was defined as hard contact in the normal direction. The separation between the profile and the die was allowed, which prevented the suppression of cure-related shrinkage. After exiting the die, which corresponds to 400 s after the initial entrance for a 150 mm/min pulling speed and a 1 m die, cooling of the profile was simulated as convective heat transfer towards the outer surroundings. The ambient temperature was defined as 20 °C, where the convection coefficient was defined as 10 W/(m${}^{2}$ K). The simulation was stopped when the profile was cooled down to 20 °C.

A set of subroutines were used to implement the material behavior into the process model. The elastic modulus evolution was defined in UMAT. USDFLD was used to import the $V_{f}$ distribution into the ABAQUS model. UEXPAN was used to define the $V_{f}$ dependent coefficient of thermal expansion and cure shrinkage. The heat generation through exothermic cure reaction depending on $V_{f}$ was included via HETVAL. $V_{f}$ dependent specific heat capacity, thermal conductivity, and density were defined in the user interface in the form of tabular data in which the $V_{f}$ was taken as a field variable. In total, 4080 elements were used in the model, which corresponds to approximately 64×64 elements through the cross-section. The mesh structure used in this study can be seen in Figure 4. The total number of elements was same for each RVE size case, i.e., the mesh density was same for each model. The element type was set to ‘8-node generalized plane strain thermally coupled quadrilateral, biquadratic displacement, bilinear temperature, hybrid, linear pressure, reduced integration’ (CPEG8RHT).

## 4. Results and Discussion

### 4.1. Fiber Distribution through the Cross-Section

**Figure 5:**
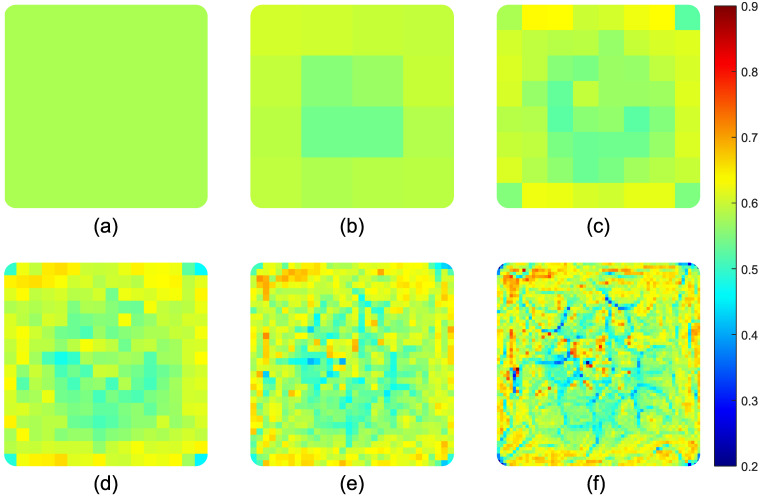
$V_{f}$ distribution through the cross-section for various RVE sizes; (**a**) uniform, (**b**) ‘Case A’ (4×4), (**c**) ‘Case B’ (8×8), (**d**) ‘Case C’ (16×16), (**e**) ‘Case D’ (32×32), (**f**) ‘Case E’ (64×64).

The estimated $V_{f}$ distribution maps for different RVE sizes are depicted in Figure 5. The average fiber radius is 14.5 μm with a standard deviation of 1.5 μm. The smallest RVE edge length used in this analysis is 312.5 μm, 22 times larger than the fiber radius. In accordance with the literature, this RVE size is large enough to represent the local mechanical properties [46]. The $V_{f}$ distributions show that there is a $V_{f}$ gradient through the cross-section, with increasing fiber content towards the center of the profile. A similar observation was reported before in the literature for a profile manufactured using the resin injection pultrusion process [38].

**Figure 6:**
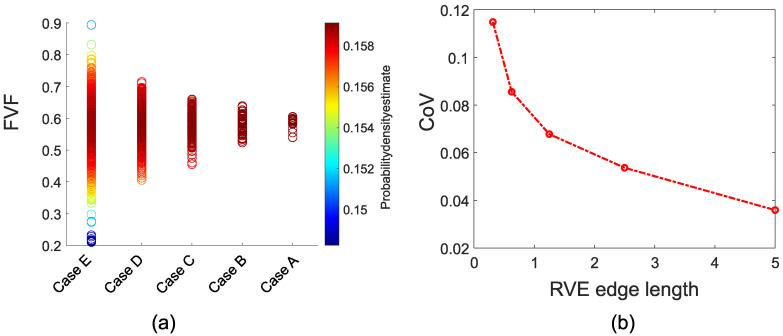
(**a**) $V_{f}$ values distribution, and (**b**) coefficient of variation with respect to the RVE edge length.

The average $V_{f}$ value through the cross-section of the profile is 58%. The overall span and the variation of the corresponding $V_{f}$ values for RVEs increase with the decreasing RVE size [47]. The minimum $V_{f}$ values for ‘Case E’ and ‘Case A’ are 21.0 and 54.0%, respectively. The maximum $V_{f}$ values are 89.4 and 60.5% respectively for the smallest and the largest windows. The coefficient of variation reduces from 11.48 to 3.59% for ‘Case E’ to ‘Case A’. The distribution of $V_{f}$ values and the coefficient of variation for different RVE sizes can be seen in Figure 6.

### 4.2. Effect of Nonuniformity on the Temperature and Cure Degree Evolution

In a conventional pultrusion process, the profile is heated from the outside via conduction from the pultrusion die. Consequently, the temperature rises at the profile’s outer regions first and then in the core region. In addition to the heating die, internal heat generation occurs during the exothermic cure reaction, which affects the thermal history, particularly in the core of the profile. In the thinner profiles, the temperature distribution can be almost uniform throughout the cross-section [15]. On the other hand, for thicker profiles, it is possible to have a more non-uniform temperature distribution due to the exothermic heat reaction. With the internal heat generation’s help, the temperature at the center of the profile can overshoot and reach a higher temperature than the die temperature. This macro-scale temperature nonuniformity is related to the process parameters and exists profiles with a uniform fiber distribution [15].

**Figure 7:**
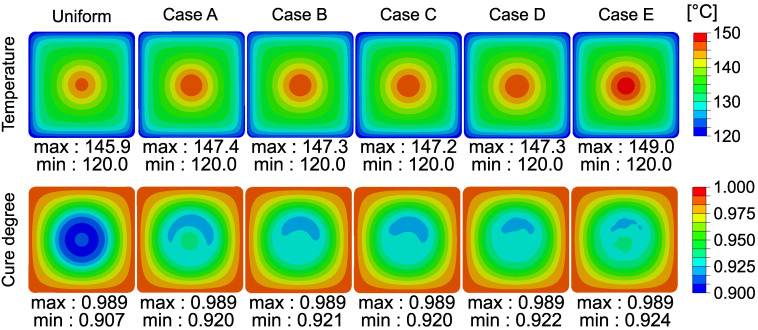
Temperature (**top row**) and cure degree (**bottom row**) distributions throughout the cross-section at the exit of the die.

The thermal conductivity of glass fiber is higher than the conductivity of the polyester resin system. It is vice versa for the specific heat capacity. With increasing fiber content, the equivalent thermal conductivity and specific heat capacity of an RVE increases and decreases, respectively [44]. Consequently, the higher fiber content at the profile’s outer regions enhances the heat transfer from the die to the core of the profile. On the other hand, a higher fiber volume content means lower exothermic heat generation due to lower resin content. These phenomena influence the overall thermal history of the profile during the process. Temperature distributions through the cross-section at the exit of the die for different window sizes are shown in Figure 7. At the die exit, the maximum temperature of the uniform case and ‘Case E’ were 145.9 and 149 °C, respectively. Throughout the process, the maximum temperatures observed in these extreme cases were 146.1 and 149.6 °C, respectively. Therefore, it can be said that the experimentally observed nonuniformity has a negligible effect on the temperature distribution. Temperature evolution through the process is strongly affected by the mold temperature, geometry, resin kinetics, pulling speed. Still, it can be said that the maximum temperature values predicted in this work were in the range of the reported values (∼140–170 °C)in the literature for polyester resin [7,48,49].

Cure degree evolution throughout the process depends on the thermal history as given in Equation (4). Here, a higher heat generation due to lower fiber content amplifies the local cure degree evolution. The cure degree distributions at the exit of the die can be seen in Figure 7. The nonuniformity had a more prominent effect on the cure degree distribution than the temperature distribution. The RVEs with relatively higher fiber content concerning the adjacent RVEs at the core region had a lower cure degree. This phenomenon can be attributed to the difference in local internal heat generation. It is worth noting that the minimum cure degree value within the profile for each case was 0.99 at the end of the process (after cool-down). At this cure degree value, the profile can be considered to be fully cured.

### 4.3. Effect of Nonuniformity on Global Residual Stress Distribution

**Figure 8:**
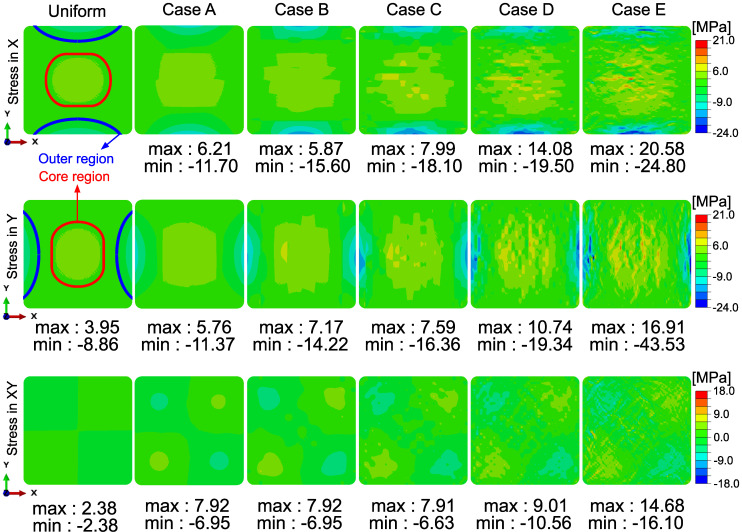
Residual stress distribution (**top row**; normal stress in X-direction, **middle row**; normal stress in Y-direction, **bottom** row; shear stress (XY)) after cooling down.

Residual stress formation in a composite material is a consequence of the thermo– chemical–mechanical history of the material itself and external sources. Therefore, the residual stress field reveals the combined effects of the temperature, cure degree, and elastic modulus evolution through the process. The predicted stress fields in the transverse plane for the process conditions presented in this study are shown in Figure 8. A similar trend was reported in the literature for square profiles as tensile residual stress in the core and the compressive at the outer sections [15,16,49]. In each RVE size case in this study, the core region was mainly in tension, which was equilibrated with compressive stresses at the outer region. However, the local stress values were scattered with decreasing window size. In addition to the observed scatter, local maximum and minimum stress values within the profile also increased with decreasing window size. The maximum tensile residual stress value observed in the ‘Case E’ was 20.58 MPa, whereas it was 3.95 MPa for the uniform case. Similarly, the maximum compressive stress predicted within the cross-section was −43.53 MPa, whereas it was −8.86 MPa in the uniform case. The increasing trend of the stress values with decreasing RVE size can be seen in Figure 8.

The boundaries of the core and outer regions were defined to compare the average stresses between different cases. The area where the stress was higher than 1.5 MPa in the uniform case was labeled as the “core region”. The area where the stress was lower than −2.0 MPa was labeled as the “outer region”. The corresponding core and outer regions are depicted in Figure 8. These areas were defined separately for the X and Y directions. The average stress and the $V_{f}$ values in the corresponding areas for different patch sizes are summarized in Table 5. The corresponding average $V_{f}$ values can be seen in parentheses.

The average $V_{f}$ values relatively low for the core, and high for the outer regions. Together with the temperature and cure degree nonuniformity, which is mainly caused by the nature of the process as seen in Figure 7, relatively higher $V_{f}$ at the outer region has an amplifying effect on the residual stress. This is because a higher $V_{f}$ increases the elastic modulus, resulting in higher stiffness at the outer region during the process [45]. On the other hand, relatively lower $V_{f}$ at the core region means higher thermal contraction during cooling and higher cure shrinkage due to polymerization of unsaturated polyester resin. Due to the outside-in heating of the profile, the core region cures later than the outer regions. Therefore, the stiffer outer shell and higher thermo–chemical contraction in the non-uniform $V_{f}$ cases result in higher average tensile stress at the core compared to the uniform $V_{f}$ case. As tabulated in Table 5, the average $V_{f}$ decreased from 0.58 to 0.55 from the uniform case to non-uniform cases. It is worth pointing out that the predicted average residual stress at the core region showed a sudden increase from the uniform case to ‘Case A’. The average tensile stress value at the core was almost equal for each non-uniform $V_{f}$ case. This sudden increase and stabilization after ‘Case A’ in the average predicted stress indicates the effect of global nonuniformity on the average tensile stress in the core. The compressive average stress values within the outer regions were converged after ‘Case B’, where the $V_{f}$ value is stabilized too. Moreover, slight differences between the average stress values in X and Y directions were caused by the asymmetry in the $V_{f}$ distribution with respect to the center axis.

### 4.4. Local Residual Stresses

**Figure 9:**
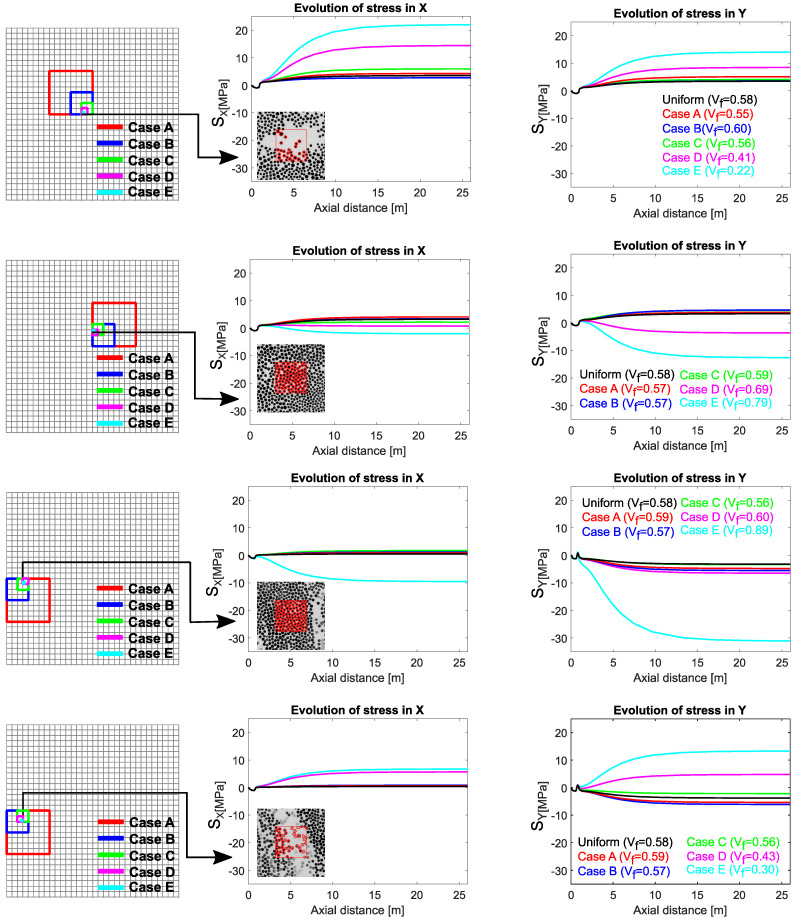
Local residual stress evolution within the selected RVEs. Schematic representations of the corresponding locations in the cross-section are on the left column (the grid is representative for ‘Case D’). Stress evolution curves in the X-direction are on the middle column. Stress evolution curves in the Y-direction are on the right column.

The stress evolution within the selected RVEs was analyzed in detail to discuss the effect of local variability in $V_{f}$ on the residual stress formation. Two RVEs from the core region and two RVEs from the outer region were selected as the comparison cases. One out of these two RVEs at the core and outer regions is in a relatively highly packed position (high $V_{f}$), and the other one is in a position with relatively lower $V_{f}$. They were picked up from the locations closer to each other to minimize the location dependent process-induced stress difference between them. These RVE locations on the cross-section and the corresponding stress evolution in the X and Y directions within these RVEs are shown in Figure 9. The stress evolution curves at the same integration points are plotted for each case to reveal the effect of RVE size on the local stresses.

The first two rows in Figure 9 show the stress evolution within the RVEs located in the core region. At the core, process-induced stresses are supposed to be in tension. The $V_{f}$ of the first RVE was 0.22 for ‘Case E’ due to a local resin pocket, as shown in the corresponding micro-graph. The $V_{f}$ of the second RVE was 0.79 for ‘Case E’, which is a highly packed RVE. For the uniform case, integration points in both RVEs had similar stress in both directions, which were around 3.3 MPa. Stress evolution in the RVE with the lower $V_{f}$ shows that the predicted stress value increases with decreasing patch size. Still, there were exceptions such as ‘Case A’ and ‘Case B’. The predicted stress value for the former case was higher than the latter despite the larger RVE size. This can be explained with the estimated $V_{f}$ values of these RVEs.

Another critical aspect in the first two rows of Figure 9 is the difference between the predicted stress values in the X and Y directions. This difference was related to the anisotropy in the neighbor RVEs’ $V_{f}$ values. On the other hand, stress evolution in an RVE with relatively high $V_{f}$ shows that the predicted stress values may even revert to compression as the patch size decreases. As a general explanation of the stress evolution within these two RVEs in the core region, a relatively low $V_{f}$ value results in enhancing the final tensile residual stress locally. On the other hand, a higher local $V_{f}$ value introduces compression to the final stress state.

Similarly, the local stress evolution at the outer region is shown in Figure 9. For the uniform distribution case, both integration points present at the depicted locations had almost zero stress in the X-direction since they are close to a free edge. In the uniform case, both have compressive stress in the Y-direction, which can be considered to be the equilibrating component of the tensile stress at the core. Stress evolution trends with decreasing RVE size in Figure 9 showed similar behavior as in the core. In other words, in a highly packed RVE, the predicted stress value increased in compression with decreasing window size.

On the other hand, stress evolution of the RVE shown at the bottom row in Figure 9, which has relatively lower fiber content due to a resin-rich layer, turned out to be in tension. The results indicate that relatively lower $V_{f}$ alters the stress value in tension, and higher $V_{f}$ alters the local stress in compression, in general. Stress values mainly differ during cooling, as shown in Figure 9. Therefore, the relation between $V_{f}$ and the process-induced stress can be attributed to the thermal expansion coefficient, which is directly related to $V_{f}$ and dominant on the thermal strain components during cooling.

**Figure 10:**
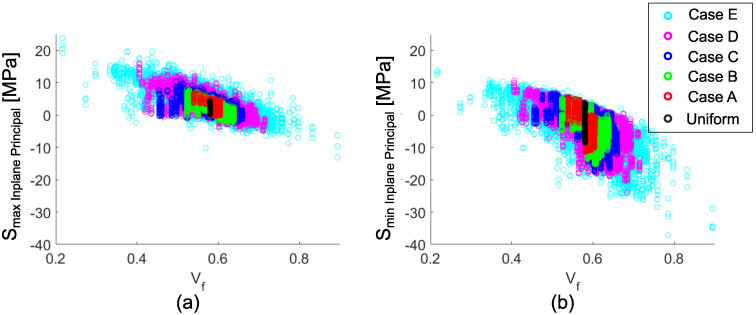
Scatter plots of the in-plane principal stresses with respect to $V_{f}$ for each patch size cases; (**a**) maximum in-plane stress, (**b**) minimum in-plane stress.

The predicted in-plane principal stress values are shown in scatter plots for each case in Figure 10 to reveal the correlation between $V_{f}$ and the predicted values throughout the cross-section. The general trends in stress distribution with respect to $V_{f}$ are similar for each case. However, a broader range of $V_{f}$ values for smaller RVE sizes results in higher stress values. The limit values for ‘Case E’ were around 24 MPa and −38 MPa, which corresponded to 21% and 89% fiber contents, respectively. The measured transverse tensile strength for glass fiber-reinforced polyester pultruded profiles are at a level of 50 MPa, and the compressive strength values are at a level of 70 MPa in the literature [50,51]. The predicted values are near critical levels. Therefore, the residual stresses should be taken into account when evaluating the performance of pultruded profiles. Moreover, here we have a location dependent $V_{f}$ gradient instead of a random distribution, which would play an essential role in the critical locations depends on the loading scenario.

### 4.5. Processing–Structure–Property Relationship

Investigating the reasons behind the $V_{f}$ gradient throughout the cross-section of this particular pultruded profile is not within the scope of this study. Still, this formation can be claimed to be formed because of the processing steps. Compaction from the outer edges at the beginning of the die can be considered one of the possible reasons for this gradient. In the end, this experimentally observed $V_{f}$ gradient promotes the tensile residual stress at the core due to relatively lower $V_{f}$ values. Similarly, this gradient promotes the compressive residual stress at the outer regions due to relatively higher $V_{f}$ as explained for the local stress evolution in Figure 9. From a structural aspect, the $V_{f}$ value affects the failure behavior of composites. Therefore, superposed effects of the location dependent residual stress and loading-induced stresses, both being related to the location dependent $V_{f}$, would be decisive on the mechanical performance.

**Figure 11:**
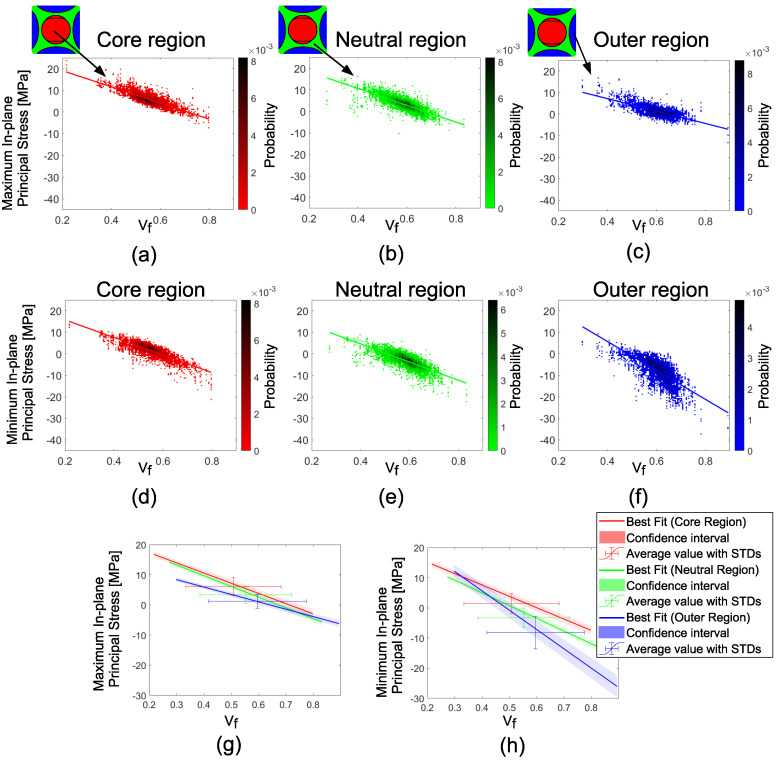
Maximum in-plane principal stresses vs. $V_{f}$ for the core (**a**), the neutral (**b**) and the outer regions (**c**). The minimum in-plane principal stresses vs. $V_{f}$ for the core (**d**), the neutral (**e**) and the outer regions (**f**). Best linear fits and confidence intervals of the maximum (**g**) and the minimum (**h**) in-plane principal stresses.

The predicted maximum and minimum in-plane principal stress distributions on different locations through the cross-section, namely the core, neutral and outer regions, are depicted in Figure 11 for ‘Case E’ to emphasize the processing–structure–property relationships. The previously defined core and outer regions, shown in Figure 8, were defined separately for X and Y components to see the direction dependent average stress predictions. These regions were united as depicted in Figure 11 for the in-plane principal stress analysis. The core region is colored red, the neutral region is colored green, and the outer region is colored blue in Figure 11. The slopes of the mean lines of maximum and minimum in-plane principal stresses were similar for the core and the neutral region, as shown in Figure 11a,b,d,e. However, the slope differentiated in the outer region as depicted in Figure 11c,f. The mean line comparisons in Figure 11g,h clearly show that the average $V_{f}$ value and residual stress values deviated for different regions. The critical regions for tension are primarily located in the core region, and the critical regions for compression are mostly located in the outer regions.

## 5. Conclusions

In this study, we investigated the $V_{f}$ distribution in a thick UD fiber-reinforced pultruded profile and its effects on the residual stress formation. These were done by characterizing the cross-sectional $V_{f}$ distribution with optical light microscopy and using the local $V_{f}$ dependent material behavior in a 2D numerical process model developed for mesoscale thermo– chemical– mechanical modeling of pultrusion.

The optical light microscopy analysis revealed that the profile had a lower fiber content in the core compared to the outer region. When we used this information in the mesoscale model, the magnitude of the overall residual stress in the core (tension) and the outer region (compression) increased more than 50%.

By implementation of the full field $V_{f}$ nonuniformity in the mesoscale, we also captured the scatteredness in the residual stress field. The stress field appeared more scattered when the RVE size became smaller. In some areas, the stress values even shifted from tension to compression, but overall, the local regions with high fiber content appeared to exhibit more compressive residual stress.

We exemplified the importance of analyzing the processing–structure–property relationship for a pultruded composite profile in this study. In conclusion, the results underline that the coupled nature of the local fiber volume fraction and the residual stress formation can be of critical importance when accessing the structural integrity of pultruded profiles. As, a huge effort is put on simulating the structural performance of pultruded composites [52]. We shed a light on the mesoscale nonuniformity in Vf and its effects on residual stress field, which can be further used for the structural analysis in the presence of this mesoscale nonuniformity.

The proposed image analysis and coupling with the thermo– chemical– mechanical model might not be directly used for pultruded composites consisting of continuous filament mat or woven fabrics. Hence, a 3D microstructural analysis and a modification in the proposed process simulation would be necessary for those types of reinforcements.

The mesoscale process modeling scheme developed in this study can also be used in a multiscale process modeling framework in future studies. The location dependent spatial fiber distribution can be implemented into advanced microscale methods to estimate the fiber distribution dependent thermal [53] and mechanical [46] properties which can be used in a multiscale process modeling scheme. Location dependent coupling between micro- meso- macro levels in a multi-scale analysis would give valuable insight into pultruded profiles’ structural limits.

## Figures and Tables

**Table 1:** The used labels, the corresponding number of patches, and the RVE edge lengths.

Label	Number of Patches	RVE Edge Length
Case A	4×4	5 mm
Case B	8×8	2.5 mm
Case C	16×16	1.25 mm
Case D	32×32	0.625 mm
Case E	64×64	0.3125 mm

**Table 2:** Cure kinetics parameters (reproduced from [41] with the permission).

$A_{0}$ [1/s]	$E_{a}$ [kJ/mol]	*m*	*n*
$7.558\times 10^{9}$	82.727	0.630	1.847

**Table 3:** Parameters used in the CHILE model to characterize the instantaneous elastic modulus of the resin system (reproduced from [41] with the permission).

$E_{r}^{0}$ [GPa]	$E_{r}^{1}$ [GPa]	$E_{r}^{\infty }$ [GPa]	$T_{C0}\;[{}^{\circ}C]$	$T_{C1}\;[{}^{\circ}C]$	$T_{C2}\;[{}^{\circ}C]$	$A_{e}$ [GPa]	$K_{e}\;[1/{}^{\circ}C]$
0.0195	0.73	3.76	−60	30	110	0.20	0.043

**Table 4:** Thermal and mechanical material properties of the glass fiber and resin system used in this study (reproduced from [7] with the permission).

	ρ [kg/m ${}^{3}$]	$C_{p}$ [J/ (kg · K)]	*k* [W/ (m · K)]	*E* [GPa]	ν	CTE [ppm/°C]
Polyester resin	1100	1830	0.17	Table	0.40	72–180
Glass fiber	2560	670	11.4 (axial) , 1.04 (transverse)	73	0.22	5.04

**Table 5:** Average stress and $V_{f}$ values in the core (tensile) and the outer regions (compressive).

	Uniform	Case A	Case B	Case C	Case D	Case E
Stress [MPa] in X @core ($V_{f}$)	2.53(0.58)	3.83(0.55)	3.84(0.55)	3.89(0.55)	3.86(0.55)	3.87(0.55)
Stress [MPa] in Y @core ($V_{f}$)	2.53(0.58)	3.82(0.55)	4.01(0.55)	4.12(0.55)	4.07(0.55)	4.10(0.55)
Stress [MPa] in X @outer ($V_{f}$)	−4.28(0.58)	−5.51(0.59)	−7.11(0.62)	−7.29(0.62)	−7.39(0.62)	−7.48(0.61)
Stress [MPa] in Y @outer ($V_{f}$)	−4.28(0.58)	−5.52(0.59)	−6.90(0.60)	−7.16(0.60)	−7.16(0.60)	−7.29(0.60)

## Data Availability

The data presented in this study are available on request from the corresponding authors.

## Abbreviations

The following abbreviations are used in this manuscript:

$V_{f}$	Fiber volume fraction
UD	Unidirectional
RVE	Representative volume element
FRPC	Fiber-reinforced polymer composite
CHILE	Cure hardening instantaneous linear elastic
CTE	Coefficient of thermal expansion
SCFM	Self-consistent field micromechanics
$T_{g}$	Glass transition temperature
1D, 2D, 3D	1-dimensional, 2-dimensional, 3-dimensional
$\Delta \epsilon_{ij}^{mech}$	Mechanical strain increment
$\Delta \epsilon_{ij}^{tot}$	Total strain increment
$\Delta \epsilon_{ij}^{therm}$	Thermal strain increment
$\Delta \epsilon_{ij}^{chem}$	Chemical strain increments
$\Delta \sigma_{ij}$	Incremental stress component
*J*	Jacobian matrix
ρ	Density
$C_{p}$	Specific heat capacity
*k*	Thermal conductivity
*E*	Elastic modulus
ν	Poisson’s ratio
$A_{0}$	Pre-exponential constant
$E_{a}$	Activation energy
*m*, *n*	Reaction orders
α	Instantaneous cure degree
*R*	Gas constant
*T*	Absolute temperature
$k_{X}$, $k_{Y}$	Thermal conductivity of composite in X and Y directions
$k_{f}$	Thermal conductivity of fiber
$k_{r}$	Thermal conductivity of resin
$\omega_{f}$	Weight fraction of fiber
$\omega_{r}$	Weight fraction of resin
$T_{C1}$, $T_{C2}$, $T_{C3}$	Critical temperatures for elastic modulus model
$E_{r}^{0}$, $E_{r}^{1}$, $E_{r}^{\infty }$	Critical elastic moduli for elastic modulus model
$A_{e}$, $K_{e}$	Exponential rise constants in elastic modulus model

## References

[1] Chung D. (2017). Processing-structure-property relationships of continuous carbon fiber polymer-matrix composites. Mater. Sci. Eng. Rep..

[2] Baran I., Cinar K., Ersoy N., Akkerman R., Hattel J. (2017). A Review on the Mechanical Modeling of Composite Manufacturing Processes. Arch. Comput. Methods Eng..

[3] Wisnom M., Gigliotti M., Ersoy N., Campbell M., Potter K. (2006). Mechanisms generating residual stresses and distortion during manufacture of polymer–matrix composite structures. Compos. Part Appl. Sci. Manuf..

[4] Vedernikov A., Safonov A., Tucci F., Carlone P., Akhatov I. (2020). Pultruded materials and structures: A review. J. Compos. Mater..

[5] Sandberg M., Yuksel O., Comminal R.B., Sonne M.R., Jabbari M., Larsen M., Salling F.B., Baran I., Spangenberg J., Hattel J.H., Silberschmidt V.V. (2020). 6-Numerical modeling of the mechanics of pultrusion. Mechanics of Materials in Modern Manufacturing Methods and Processing Techniques.

[6] Baran I., Hattel J.H., Akkerman R. (2015). Investigation of process induced warpage for pultrusion of a rectangular hollow profile. Compos. Part Eng..

[7] Baran I., Akkerman R., Hattel J.H. (2014). Modelling the pultrusion process of an industrial L-shaped composite profile. Compos. Struct..

[8] Baran I. (2015). Pultrusion: State-of-the-Art Process Models.

[9] Safonov A., Gusev M., Saratov A., Konstantinov A., Sergeichev I., Konev S., Gusev S., Akhatov I. (2020). Modeling of cracking during pultrusion of large-size profiles. Compos. Struct..

[10] Batch G.L., Macosko C.W. (1993). Heat transfer and cure in pultrusion: Model and experimental verification. AIChE J..

[11] Gorthala R., Roux J.A., Vaughan J.G. (1994). Resin Flow, Cure and Heat Transfer Analysis for Pultrusion Process. J. Compos. Mater..

[12] Carlone P., Palazzo G., Pasquino R. (2006). Pultrusion manufacturing process development by computational modelling and methods. Math. Comput. Model..

[13] Baran I., Tutum C.C., Hattel J.H. (2013). The effect of thermal contact resistance on the thermosetting pultrusion process. Compos. Part Eng..

[14] Sandberg M., Yuksel O., Baran I., Hattel J.H., Spangenberg J. (2020). Numerical and experimental analysis of resin-flow, heat-transfer, and cure in a resin-injection pultrusion process. Compos. Part Appl. Sci. Manuf..

[15] Baran I., Tutum C.C., Nielsen M.W., Hattel J.H. (2013). Process induced residual stresses and distortions in pultrusion. Compos. Part Eng..

[16] Baran I., Hattel J., Akkerman R., Tutum C. (2015). Mechanical Modelling of Pultrusion Process: 2D and 3D Numerical Approaches. Appl. Compos. Mater..

[17] Sandberg M., Yuksel O., Baran I., Spangenberg J., Hattel J.H. (2021). Steady-state modelling and analysis of process-induced stress and deformation in thermoset pultrusion processes. Compos. Part Eng..

[18] Baran I., Tutum C., Hattel J., Akkerman R. (2015). Pultrusion of a vertical axis wind turbine blade part-I: 3D thermo-chemical process simulation. Int. J. Mater. Form..

[19] Struzziero G., Maistros G., Hartley J., Skordos A. (2021). Materials modelling and process simulation of the pultrusion of curved parts. Compos. Part Appl. Sci. Manuf..

[20] Vedernikov A., Tucci F., Carlone P., Gusev S., Konev S., Firsov D., Akhatov I., Safonov A. (2021). Effects of pulling speed on structural performance of L-shaped pultruded profiles. Compos. Struct..

[21] Yuksel O., Baran I., Ersoy N., Akkerman R. (2019). Investigation of transverse residual stresses in a thick pultruded composite using digital image correlation with hole drilling. Compos. Struct..

[22] Yuksel O., Baran I., Ersoy N., Akkerman R. (2018). Analysis of residual transverse stresses in a thick UD glass/polyester pultruded profile using hole drilling with strain gage and digital image correlation. AIP Conf. Proc..

[23] Mesogitis T., Skordos A., Long A. (2014). Uncertainty in the manufacturing of fibrous thermosetting composites: A review. Compos. Part Appl. Sci. Manuf..

[24] Potter K., Khan B., Wisnom M., Bell T., Stevens J. (2008). Variability, fibre waviness and misalignment in the determination of the properties of composite materials and structures. Compos. Part Appl. Sci. Manuf..

[25] Haanappel S., ten Thije R., Sachs U., Rietman B., Akkerman R. (2014). Formability analyses of uni-directional and textile reinforced thermoplastics. Compos. Part Appl. Sci. Manuf..

[26] Krämer E., Grouve W., Koussios S., Warnet L., Akkerman R. (2020). Real-time observation of waviness formation during C/PEEK consolidation. Compos. Part Appl. Sci. Manuf..

[27] Matveev M., Belnoue J.H., Nixon-Pearson O., Ivanov D., Long A., Hallett S., Jones I. (2019). A numerical study of variability in the manufacturing process of thick composite parts. Compos. Struct..

[28] David A., Darrow J., Smith L.V. (2002). Isolating Components of Processing Induced Warpage in Laminated Composites. J. Compos. Mater..

[29] Çınar K., Ersoy N. (2015). Effect of fibre wrinkling to the spring-in behaviour of L-shaped composite materials. Compos. Part Appl. Sci. Manuf..

[30] Paciornik S., Martinho F., de Mauricio M., d’Almeida J. (2003). Analysis of the mechanical behavior and characterization of pultruded glass fiber–resin matrix composites. Compos. Sci. Technol..

[31] Rasmussen F., Sonne M., Larsen M., Mikkelsen L., Hattel J. A Characterization Study Relating Cross-Sectional Distribution of Fiber Volume Fraction and Permeability. Proceedings of the 22nd International Conference on Composite Materials 2019, ICMM22.

[32] Morales C.N., Claure G., Álvarez J., Nanni A. (2020). Evaluation of fiber content in GFRP bars using digital image processing. Compos. Part Eng..

[33] Carlone P., Baran I., Hattel J.H., Palazzo G. (2013). Computational approaches for modeling the multiphysics in pultrusion process. Adv. Mech. Eng..

[34] Kratmann K., Sutcliffe M., Lilleheden L., Pyrz R., Thomsen O. (2009). A novel image analysis procedure for measuring fibre misalignment in unidirectional fibre composites. Compos. Sci. Technol..

[35] Poulton M., Sebastian W. (2021). Taxonomy of fibre mat misalignments in pultruded GFRP bridge decks. Compos. Part Appl. Sci. Manuf..

[36] Baran I., Straumit I., Shishkina O., Lomov S.V. (2018). X-ray computed tomography characterization of manufacturing induced defects in a glass/polyester pultruded profile. Compos. Struct..

[37] Sebastian W.M. (2018). Fibre waviness in pultruded bridge deck profiles: Geometric characterisation and consequences on ultimate behaviour. Compos. Part Eng..

[38] Rasmussen F., Klingaa C., Larsen M., Sonne M., Spangenberg J., Hattel J. Modelling the Effect of Non-Uniform Fibre Distribution on the Curing Behaviour in Resin Injection Pultrusion. Proceedings of the 18th European Conference on Composite Materials (ECCM-18).

[39] Baran I. (2017). Analysis of the local fiber volume fraction variation in pultrusion process. Proceedings of the 20th International ESAFORM Conference on Material Forming.

[40] Yuksel O., Baran I., Rasmussen F., Spangenberg J., Ersoy N., Hattel J., Akkerman R. Meso-Scale Process Modelling Strategies for Pultrusion of Unidirectional Profiles. Proceedings of the 18th European Conference on Composite Materials (ECCM-18).

[41] Baran I., Akkerman R., Hattel J.H. (2014). Material characterization of a polyester resin system for the pultrusion process. Compos. Part Eng..

[42] Yuksel O., Sandberg M., Baran I., Ersoy N., Hattel J.H., Akkerman R. (2021). Material characterization of a pultrusion specific and highly reactive polyurethane resin system: Elastic modulus, rheology, and reaction kinetics. Compos. Part Eng..

[43] Khoun L., de Oliveira R., Michaud V., Hubert P. (2011). Investigation of process-induced strains development by fibre Bragg grating sensors in resin transfer moulded composites. Compos. Part Appl. Sci. Manuf..

[44] Lin R.J., Lee L.J., Liou M.J. (1993). Mold filling and curing analysis in liquid composite molding. Polym. Compos..

[45] Bogetti T.A., John W. (1992). Gillespie, J. Process-Induced Stress and Deformation in Thick-Section Thermoset Composite Laminates. J. Compos. Mater..

[46] Bhuiyan F.H., Sanei S.H.R., Fertig R.S. (2020). Predicting variability in transverse effective elastic moduli and failure initiation strengths in UD composite microstructures due to randomness in fiber location and morphology. Compos. Struct..

[47] Sanei S.H.R., Fertig R.S. (2015). Uncorrelated volume element for stochastic modeling of microstructures based on local fiber volume fraction variation. Compos. Sci. Technol..

[48] Han C.D., Chin H.B. (1988). Development of a mathematical model for the pultrusion of unsaturated polyester resin. Polym. Eng. Sci..

[49] Baran I. (2016). Analysis of pultrusion process for thick glass/polyester composites: Transverse shear stress formations. Adv. Manuf. Polym. Compos. Sci..

[50] Carra G., Carvelli V. (2014). Ageing of pultruded glass fibre reinforced polymer composites exposed to combined environmental agents. Compos. Struct..

[51] Zhang L., Liu W., Wang L., Ling Z. (2020). On-axis and off-axis compressive behavior of pultruded GFRP composites at elevated temperatures. Compos. Struct..

[52] Fascetti A., Feo L., Abbaszadeh H. (2021). A critical review of numerical methods for the simulation of pultruded fiber-reinforced structural elements. Compos. Struct..

[53] Leonid B., Alexander G., Kolpakov A.N. (2013). Introduction to the Network Approximation Method for Materials Modeling.

